# Candida Albicans Osteomyelitis after Chest Wall Blunt Trauma: A Case Report

**DOI:** 10.1155/2021/9987317

**Published:** 2021-06-03

**Authors:** Fabrizio Minervini, Peter B. Kestenholz, Elmar Fritsche, Alberto Franchi

**Affiliations:** ^1^Department of Thoracic Surgery, Kantonsspital Luzern, Lucerne, Switzerland; ^2^Department of Plastic Surgery, Kantonsspital Luzern, Lucerne, Switzerland

## Abstract

Fungal osteomyelitis is a rare disease that can occur in immunocompromised patients. We report a case of a patient with a primary rib osteomyelitis after a blunt trauma of the chest wall. Aggressive surgical debridement along with antifungal therapy was the cornerstone of the disease management in this patient.

## 1. Introduction

The most common type of injury after a blunt chest wall trauma is represented by rib fractures, which are associated with significant morbidity and mortality due to respiratory complications deriving from pain and an impaired ventilation capacity [1].

According to a recent systematic review, a wound infection can occur as a complication in about 2.2% of those patients who underwent surgical ribs stabilization after rib fractures [2]. On the contrary, a soft tissue/bone infection following conservative management of rib fractures is a rarely reported condition [3].

## 2. Case Presentation

A 71-year-old immunocompetent Caucasian male patient presented to the emergency department via ambulance a few hours after a fall from a height of 3 meters. After a primary survey, a computed tomography (CT) imaging revealed clavicle fracture, serial rib fractures associated with pulmonary contusion and hemopneumothorax on the left side, and non-displaced serial rib fractures on the right side.

He underwent surgery with surgical stabilization with plates of the clavicle and the ribs on the left side and was discharged home on the 19th postoperative day (POD).

Three months later, he presented with a painful swelling on his right chest wall without a history of new trauma or surgery on that side. Physical examination showed a large solitary swelling over the seventh/eighth rib, 3 × 5 cm in dimension. The lesion was soft, tender, fluctuating with defined margins, and not attached to underlying structures.

**Figure 1:**
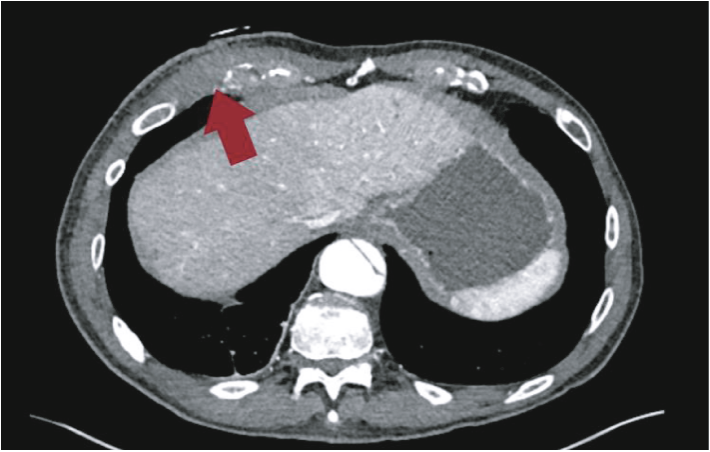
CT Scan showing fluid collection (red arrow).

**Figure 2:**
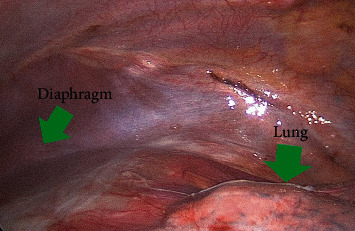
A diagnostic thoracoscopy showed no abnormal findings.

**Figure 3:**
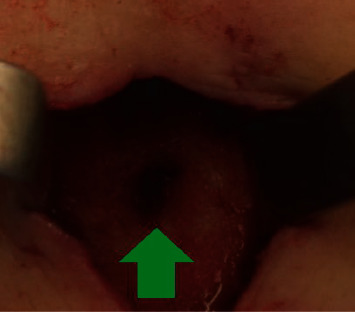
After draining the purulent collection, a hole of the 7th ribs was visualized (green arrow).

In order to determine the cause of the swelling, we performed an ultrasonography and a CT Scan that showed a capsulated fluid collection (Figure 1). A diagnostic thoracoscopy showed no abnormal intrathoracic findings (Figure 2), and therefore, an open incision of the collection was performed. Intraoperatively, we confirmed the presence of an encapsulated abscess with purulent secretion coming out from a hole in the 7th rib (Figure 3).

After partial resection of the affected rib and tissue sampling for histological and microbiological examination, a negative pressure dressing was applied (Wound V.A.C. Therapy; KCI, San Antonio, Texas).

**Figure 4:**
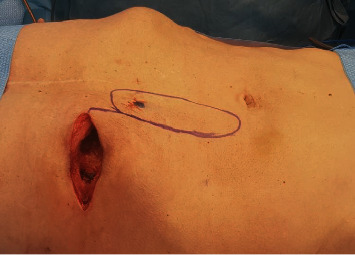
Intraoperative picture at time of reconstruction after 4 cycles of VAC Therapy. The defect at the level of the 7^th^ rib measured 10 × 6 × 4 cm. The superior epigastric artery perforator (SEAP) flap was designed with the musculocutaneous perforator reaching the skin at the proximal end of the flap (black dot).

**Figure 5:**
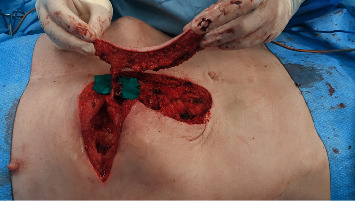
The superior epigastric artery perforator flap was harvested and the vascular pedicle (superior epigastric artery) skeletonized until the caudal margin of the 8^th^ rib to allow maximum freedom of movement and precise flap insetting.

**Figure 6:**
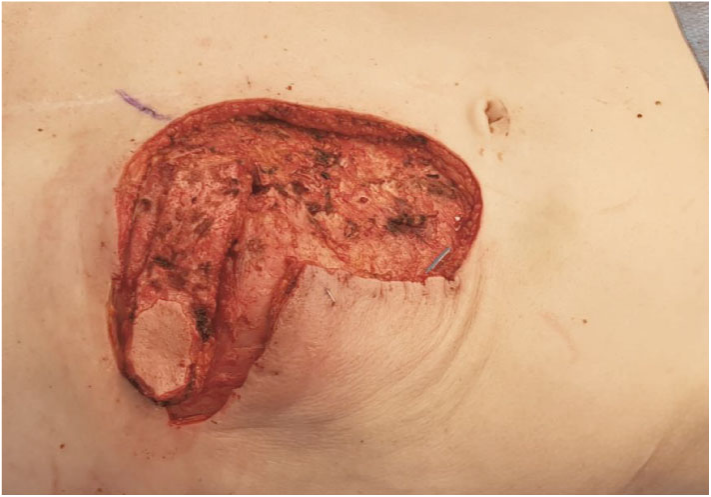
The flap was rotated by 90° and about 70% of the skin surface of the flap was deepithelialized to allow filling of the cavity as a “buried flap.” The remaining 30% served as a skin island for postoperative flap monitoring.

Histopathologic examination revealed extensive granulocyte infiltrates. The presence of Candida albicans was detected in the resected tissue but not in blood cultures (in absence of specific treatment). Dressing changes were scheduled every 3 days until a clean granulating wound without any residual sign of infection was obtained. Two weeks after the initial debridement of the infected tissue, the defect measured approximately 10 × 6 cm and was about 4 cm deep. After confirming the presence and course of a sizeable perforator originating from the superior epigastric artery in the proximity of the wound by means of a CT angiography and handheld doppler, a perforator-based superior epigastric artery perforator (SEAP) propeller flap was planned (Figure 4). After its harvest, most of the flap (about 70% of its surface) was deepithelialized and buried to adequately fill the dead space, while the distal tip of the flap served as a monitor to check its perfusion postoperatively (Figures 5 and 6).

The postoperative course was thereafter uneventful, and the patient could be discharged 7 days after the wound closure. On the day of discharge, the wound was healing without any swelling or tenderness of the affected area. At 21 days follow-up, all the wounds were healed, and the patient was allowed to return to all normal activities without any restrictions. Further surgery to remove the monitor skin island under local anesthesia was offered for cosmetic reasons, but the operation was postponed due to the COVID-19 outbreak.

**Figure 7:**
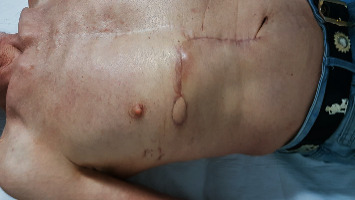
Postoperative picture at 9 months showing stable conditions. Further surgery to remove the skin island was offered.

He was treated with fluconazole for 6 months. After a 9-month follow-up, we found no evidence of infection or abnormal wound healing (Figure 7).

## 3. Discussion

Blunt thoracic injuries can potentially affect any components of the chest wall or thoracic cavity being responsible for 25% of polytrauma patient death [4].

The most prevalent type of injury following thoracic blunt trauma is represented by rib fractures which are responsible for a high pulmonary morbidity due to pain and decreased lower airway clearance.

Other pathological findings that can be potentially life-threatening conditions are pneumothorax, hemothorax, pericardial tamponade, and airway rupture.

Initial clinical assessment according to the Advanced Trauma Life Support (ATLS) protocol along with diagnostic imaging modalities (including chest X-ray, bedside ultrasound, and chest CT Scan represent a cornerstone in order to identify pathological conditions that might potentially lead to patient's death.

The diagnostic role of surgery (and particularly minimal invasive surgery in the case of hemodynamically stable patients) in the management of chest trauma should not be underestimated [5]. Video-assisted thoracoscopic surgery (VATS) as a diagnostic/therapeutic procedure for pleural space management in noncritical patients could be very helpful [6].

A diagnostic thoracoscopy, with a complete visualization of the pleural cavity, can exclude several trauma-related injuries (such as in our case), reveal easily misdiagnosed lesions (like a diaphragmatic rupture), or be useful to treat a concomitant hemothorax [7].

Following trauma, immune function can decrease due to the release of local inflammatory cytokines and thus causing more susceptibility to infections [8].

Nevertheless, infections of the chest wall in blunt thoracic trauma are a very rare entity.

Osteomyelitis is an infection of the bone caused, in the majority of cases, by the presence of bacteria. Fungal osteomyelitis is a rare condition, with Candida and Aspergillus being the common agents involved [9].

Common risk factors for fungal colonization are immunosuppression, diabetes, parenteral nutrition use of broad-spectrum antibiotics, Candida colonization, and central venous catheters. None of those was present in our case.

Gamaletsou et al. noted that although fungal osteomyelitis remains rare, as many as 10% of rib osteomyelitis cases were due to fungal pathogens, and the incidence is increasing with the increasing number of susceptible hosts [10]. However, rib osteomyelitis occurring de novo is very rare [3], and to our knowledge, no cases of primary osteomyelitis of fractured ribs are reported in the literature so far. Even if the Infectious Diseases Society of America suggests an antifungal therapy for 6-12 months possibly associated with surgery, there are no established guidelines regarding the treatment of candida osteomyelitis [11]. In our case, we performed radical debridement of the infected tissue, including partial resection of the affected 7th rib, followed by a delayed coverage of the defect with a local flap.

The pedicled, superior epigastric artery perforator (SEAP) flap is a well-known option to cover soft-tissue defects of the anterior chest wall [12, 13]. Although the intramuscular dissection of the vascular pedicle can be challenging and requires microsurgical experience, this flap allows excellent freedom of movement, accurate flap insetting, and minimal donor site morbidity. In our case, the flap was tailored, mostly deepithelialized, and buried to accurately obliterate the dead space resulted from the missing rib. Even if the flap could be completely deepithelialized and buried under the advancement skin flaps from the wound margins, we kept a skin island to allow easy monitoring of the flap perfusion, thus being able to promptly identify signs of vascular compromise (e.g., due to pedicle torsion or compression). Complete obliteration of the dead space and optimal perfusion of the reconstructive flap are of paramount importance when treating wounds with high risk of severe infection [14].

## 4. Conclusion

Primary rib fungal osteomyelitis is a rare condition, and the current knowledge about its diagnosis and management mainly comes from case reports and small series. To our knowledge, this is the first case of primary rib osteomyelitis sustained by Candida albicans in an otherwise healthy patient.

Our case was successfully managed with aggressive debridement of the infected tissue, delayed reconstruction with a local perforator-based flap, and targeted antifungal therapy.
